# Professional Status and Education

**Published:** 1877-11

**Authors:** W. W. H. Thaxton

**Affiliations:** Farmville, Va.


### PROFESSIONAL STATUS, AND EDUCATION.

BY W. W. H. THAXTON, D. D. S., FARMVILLE, VA.

*Read before the Tennessee Dental Association.*

MR. PRESIDENT AND GENTLEMEN:—

While I cannot now prepare a finished "paper," I will so far avail myself of your polite request, as to submit a few observations and suggestions in the trust that I may accomplish some little good for our common cause.

The practice, the culture, and the advancement of Dental Science, has with us been a life-long pursuit; all our energies, and whatever of ability Providence has vouchsafed us, have been enlisted in behalf of our profession, all of our highest and most cherished aspirations have centered and clustered round the "Ark" of our professional "Covenant."

We long for the day when "Dental Science" shall achieve its highest possible development, and occupy all over the world the same plane and position accorded all other acknowledged departments of science; when disenthralled from empiricism, and disentangled from all dependence for character and station, upon kindred departments and professions; it can be maintained, and taught, and practiced, and loved, and honored for itself, an for its own peculiar claims to public confidence and popular esteem; when as a sharply-defined, independent, self-sustaining, and self-perpetuating profession, Dental Surgery may challenge favorable comparison with the most elevated callings and pursuits of men.

We long for the day when dentists as a class, shall be as noted and distinguished for their scholarship, their social and literary accomplishments, and for their professional skill and ability, as are the physicians, the lawyers, the theologians or the votaries and disciples of any other science or profession. We love and honor medicine and general surgery, we know what they are, we love and honor the learning, the beneficence, and the illustrious men who have given dignity, character and renown to medicine and its annals.

But Mr. President and Gentlemen of the "Tennessee Dental Association," we are weary of living in the borrowed, or reflected light of medicine and general surgery, we are tired of playing "creeper," or "parasite" to the "medical oak," we are weary of the "blue glass" shield, with which medicine is supposed to cover what are denominated "its specialties." In other words, we are weary of the dependant and subordinate relation which, by common consent, Dental Surgery has so long sustained to medicine as a profession.

We wish to see Dental Surgery no longer a "specialty," but an independent, well-rounded, symmetrical profession—self-poised and self-sustained, growing, developing, and keeping step in the march of modern progress and improvement.

No science, no calling, and no profession can accomplish its higher development, while occupying a secondary or subordinate relation to any other science, profession, or pursuit. This is a truth we presume none will gainsay, why then shall we any longer tether dentistry to the fetlocks, or to the caudal extremity of medicine? By so doing, are we not repelling, and driving off the best talent of the age, that would otherwise adopt our profession, quicken our progress, and advance our standards?

But say some of our good men, how can we accomplish this change? Dentistry is a part of medicine, and a part of surgery, the relationship is so intimate and so blended, that we can't see how it can be modified or dissolved? The answer is, by adopting the identical means and methods, that gave to medicine its individuality, and its independent character and existence. By aggregating, appropriating, and adopting as an educational curriculum, the divisions, and departments of general science recognized, and acknowledged as essential to medical culture and training, medicine achieved its establishment as a distinct and independent profession. Dental Surgery can, and we hope is now doing the same thing. The profession of medicine, has no peculiar or exclusive right, no vested or proprietory claims upon Anatomy, Physiology, Pathology, Therapeutics, Chemistry, or any other special division or department of science. Dental Surgery has also aggregated, appropriated, and adopted these same and other necessary departments of general science, as an educational curriculum, and has established its schools and colleges, with all the chartered rights and privileges, and with all the equipment and paraphernalia requisite to the most thorough and perfect education and training for all the duties and offices of a distinct, a well defined, and most indispensable and important profession.

And this, Mr. President, brings to us the gist and kernel of our subject. The great and paramount duty of the hour is, for the profession to uphold, sustain, and if possible to endow these colleges, or at least, as many of them as are necessary to the demands of the public, for thorough professional culture and training. Let us as dentists, think not less of medicine, its schools, its literature, and its degree, but let us cherish more warmly, and honor more highly our own distinctive institutions, and the privileges, the benefits, and the dignities which they alone can confer. Let us not think less of the "M. D.," but more of the "D. D. S." which to the dentist is the more significant and honorable symbol.

It is an arrogant and ridiculous assumption, that the Medical Degree is in any sense superior, or that it covers all, and more than all that is expressed by our own chosen symbol. The "D. D. S.," expresses all in dentistry, that "M. D.," does in medicine, and whenever, and wherever, the graduated dentist is true to himself, and true to his profession, his degree will carry with it, and confer all the dignity and honor and public confidence that "M. D.," or any other title can bestow. Medical men, and society at large, are ready to recognize and acknowledge the professional dignity, and social station of such dentists as properly respect themselves, and honor their distinctive degree; but we need not expect or hope for such recognition, if we openly or tacitly acknowl dge or accept an inferior, or subordinate relation to any of the learned and honorable professions.

And now Mr. President, in concluding this very hasty and imperfect communication, let me adjure you to stand by "our altars and our fires," stand by and sustain our schools, our colleges, our own professional literature, and our professional badges and emblems. Let us be careful observers, and diligent students, let us lay under contribution the broad domain of science, and gather, and appropriate all that will add to our resources, and enrich our common treasury of knowledge. Let us continue true to ourselves and our chosen calling, and we have no fear but that we shall yet see the day, when Dental Surgery will stand upon the same plane occupied by all learned and liberal professions.

